# iTRAQ-based quantitative proteomic analysis of thoracic aortas from adult rats born to preeclamptic dams

**DOI:** 10.1186/s12014-021-09327-9

**Published:** 2021-08-21

**Authors:** Bin Yu, Hong-Dan Zhu, Xiao-Liang Shi, Pan-Pan Chen, Xiang-Mei Sun, Gui-Yu Xia, Min Fang, Yong-Xing Zhong, Xiao-Li Tang, Tao Zhang, Hai-Tao Pan

**Affiliations:** 1 Shaoxing Maternity and Child Health Care Hospital, Shaoxing, 312000 China; 2 Obstetrics and Gynecology Hospital of Shaoxing University, Shaoxing, China

**Keywords:** Preeclampsia, Offspring, iTRAQ, Gamete and Embryo-Fetal Origins of Adult Diseases, Thoracic aortic

## Abstract

**Background:**

Preeclampsia and gestational hypertension can cause vascular function impairment in offspring. In our previous work, we described the protein expression profiles of umbilical artery tissues from patients with preeclampsia.

**Methods:**

To gain insights into the mechanisms of vascular dysfunction in adult rats born to preeclamptic dams, we analyzed thoracic aorta tissues by using iTRAQ isobaric tags and 2D nano LC-MS/MS.

**Results:**

By using the iTRAQ method, we analyzed 1825 proteins, of which 106 showed significantly different expression in the thoracic aortic. Ingenuity pathway analysis (IPA) showed that the majority of differentially expressed proteins (DEPs) were associated with cardiovascular function. Further analysis indicated that glucose-6-phosphate dehydrogenase (G6PD), which is inhibited by miR-423-5p and activated by TP53, had the strongest effect on cardiovascular function. The expression of G6PD was upregulated in thoracic aorta tissues, as confirmed by Western blotting. The expression of two other vascular function-related proteins, cysteine- and glycine-rich protein 2 (CSRP2) and tubulin alpha-4 A (TUBA4A), was upregulated, as demonstrated by mass spectrometry (MS).

**Conclusions:**

Although the results require further functional validation, these data provide novel findings related to vascular function impairment in the adult offspring of preeclamptic mothers.

**Supplementary Information:**

The online version contains supplementary material available at 10.1186/s12014-021-09327-9.

## Introduction

Preeclampsia, a major complication of pregnancy, is characterized by a high incidence of fetal and maternal mortality [1]. Previous studies have defined the condition as de novo elevation of blood pressure (BP) to > 140/90 mmHg after the 20th week of gestation followed by proteinuria (> 0.3 g/24 h or ≥ + 1 on dipstick analysis) [2, 3]. Earlier studies have shown that women who develop preeclampsia are predisposed to cardiovascular diseases [4, 5]. Interestingly, recent epidemiological studies have suggested that preeclampsia affects not only the mother but also the child [6–8].

In fact, a number of reports have indicated that preeclampsia leads to an increased risk of cardiovascular disease in offspring. Geelhoed et al. [9] demonstrated that gestational hypertension and preeclampsia were correlated with elevated diastolic blood pressure (DBP) and systolic blood pressure (SBP) in 9-year-old offspring. On the other hand, Tenhola et al. [10] found that the 12-year-old offspring of preeclamptic mothers exhibited significantly elevated DBP and SBP relative to corresponding controls from normal mothers. Moreover, Seidman et al. [11] found no evidence linking maternal preeclampsia with abnormal cognitive performance or growth in late adolescent children but reported that the condition is linked to hypertension. Kajantie et al. [12] reported a significant association between preeclampsia and an increased risk of stroke and found that severe preeclampsia is also linked to the incidence of hypertension in adult offspring aged 60–70 years. However, the molecular mechanisms underlying this phenomenon remain elusive.

In our previous work, we revealed the protein profiles of umbilical artery tissue from preeclamptic patients [18]. Herein, we sought to elucidate the drivers of vascular dysfunction in adult rats born to preeclamptic dams. Specifically, we performed analysis of thoracic aorta tissues utilizing 2D nano LC-MS/MS and iTRAQ isobaric tags followed by quantitative proteomics analysis of thoracic aorta tissues from adult (1 year old) rats born to preeclamptic dams. Our findings provide new knowledge about vascular impairment in the adult rat offspring of preeclamptic dams.

## Materials and methods

### Animal model

The animal handling protocols were approved by the Zhejiang University Committee on Animal Care and Use, and the experiments adhered to the National Institutes of Health Guidelines for the Care and Use of Laboratory Animals. Adult male Sprague-Dawley rats (6- to 8-weeks-old) were procured from the Experimental Animal Center of Zhejiang University (Hangzhou, China), housed in our laboratory under standard conditions and provided free access to food and water. Adult male rats (6–8 weeks old) and female rats (6–8 weeks old) were allowed to freely mate to produce offspring for subsequent studies. The l-NAME preeclampsia model Inhibition of nitric oxide synthase with N-omega-nitro-L-arginine methyl ester (L-NAME) was used as experimental model of human preeclampsia [13–16]. Briefly, pregnant rats (6–8 weeks old) were obtained for the experiments by mating male (6–8 weeks old) and female rats (6–8 weeks old). Ten pregnant rats were divided into the following two groups: a group treated with L-NAME (50 mg/kg) and a control group treated with saline. The rats were administered L-NAME or saline via osmotic minipumps starting on gestational day 7 until day 7 postpartum. A total of 22 control pups and 24 L-NAME-treated pups were born, and the pups were weaned at 21 days. The thoracic aortas of rats from the two different groups were removed when the rats were 1 year old, and the blood was removed by rinsing. The thoracic aorta specimens were frozen at − 80 °C.

The BP of 1-year-old rats (from the same groups used for thoracic aortic sample collection) was measured via the tail-cuff method. Approximately 2-mm segments of mesenteric arteries (from the second-order branch of the superior mesenteric artery) were obtained, the fat and connective tissues were removed, and the tissues were mounted on a wire myograph chamber (DMT 620 M; Aarhus N, Denmark) according to the manufacturer’s recommendations. Briefly, the arterial vessel segments were soaked in physiological Krebs’ buffer that had been gassed with 95% oxygen/5% carbon dioxide (pH 7.4) and incubated at 37 °C. The tissues were subjected to an equilibration period for 30 min, vessel tension was elevated to 1 mN, and then resting tension was established for 30 min. High-potassium physiological solution (KPSS) was used to induce maximum contraction of the mesenteric arteries, which were then subjected to varying concentrations of the vasoconstrictor phenylephrine (Phe) (10−9 to 10−5 mol/L). The resulting contraction data are presented as the percentage of contraction induced by 120 mM KPSS. Thereafter, a stable contraction plateau was established by treating the mesenteric arterial rings with Phe (1 μm) to assess acetylcholine-induced vasodilatation. Subsequently, they were treated with varying concentrations of acetylcholine (10−8 to 10−5 mol/L). The results are displayed as the % of precontraction relative to that induced by Phe.

### Sample preparation

Approximately 100 mg of each thoracic aorta specimen was first frozen in liquid nitrogen and crushed to form a fine powder and then mixed with a buffer comprising 4% SDS, 1 mM DTT, and 150 mM Tris-HCl (pH 8.0) to extract total protein. The BCA protein assay (Pierce, Rockford, IL, USA) was used to quantify the concentrations of the extracted proteins.

### Protein digestion and iTRAQ labeling

The proteins were digested using a previously described protocol [17, 18]. Approximately 200 µg of total protein was suspended in 30 µL of the aforementioned extraction buffer supplemented with 100 mM dithiothreitol solution and heated at 95 °C for 5 min. The samples were cooled and subjected to ultrafiltration (cutoff of 10 kDa, Sartorius, Goettingen, Germany) with 200 µL UT buffer (150 mM Tris-HCl, and 8 M urea, pH 8.0). They were then subjected to centrifugation for 30 min at 14,000×*g* at 20 °C. Next, reduced cysteines were blocked with 100 µL 50 mM iodoacetamide dissolved in UT buffer, and then the samples were incubated for 20 min in darkness. The resultant filtrates were centrifuged at 14,000×*g* for 20 min at 20 °C and washed two times with 100 µL UT buffer at 14,000×*g* for 20 min. Subsequently, the filtrates were mixed with 100 µL dissolution buffer (AB Sciex, Framingham, MA, USA) and centrifuged at 14,000×*g* and 20 °C for 30 min. This procedure was carried out twice. The obtained filtrates were incubated overnight with 2 µg trypsin in 40 µL buffer at 37 °C. The filtrates were placed individually in fresh tubes and spun at 14,000×*g* at 20 °C for 30 min Then, the peptide concentrations were determined by measuring the UV light spectral density at OD_280_ [19].

We labeled the resultant peptide mixture with 8-plex iTRAQ reagent (AB Sciex, Framingham, MA, USA). Four thoracic aorta specimens from the control group (C) were labeled with iTRAQ tags with a mass of 113, 114, 115 and 116, while four corresponding preeclamptic tissues were labeled with iTRAQ tags with a mass of 117, 118, 119 and 121. The peptides were incubated with labeling solution at room temperature for 2 h before further analysis.

### Strong cation-exchange chromatography (SCX) separation

SCX was carried out according to a previous protocol [18]. Briefly, we acidified combined samples using 1% trifluoroacetic acid. Then, using a PolySULFOETHYL column (4.6 × 100 mm, 5 μm, 200 Å, Poly LC Inc., Columbia, MD, USA), the samples were subjected to SCX fractionation using the AKTA Explorer 100 system (GE Healthcare). Solvent A comprised 10 mM KH_2_PO_4_ in 25% (v/v) ACN, whereas solvent B comprised solvent A with 500 mM KCl. Solvents A and B were applied at the following gradients: 0–10% solvent B for 2 min, 10–20% solvent B for 25 min, 20–45% solvent B for 5 min, and 50–100% solvent B for 5 min. The fractions collected each minute were analyzed by measuring the absorbance at 214 nm. Finally, all samples were combined to form 10 fractions according to the quantity of peptides, and then the samples were desalted using C18 cartridges (Sigma). Each SCX salt step fraction was dried under vacuum centrifugation and then suspended in 40 µL 0.1% (v/v) trifluoroacetic acid.

### LC-ESI-MS/MS analysis

LC-ESI-MS/MS analysis was carried out with 5 *µ*g peptide mixture from each fraction analyzed using nano LC-MS/MS as described previously [18]. In brief, the peptide mixtures were loaded into a Thermo EASY-nLC column (100 mm× 75 µm, 3 µm; Thermo Finnigan, San Jose, CA, USA) in solvent C (0.1% formic acid) and separated using the following linear gradient of solvent D (80% acetonitrile with 0.1% (v/v) formic acid) at a flow rate of 300 nL/min over 120 min: 0–100 min at 0–45% solvent D, 100–108 min at 45–100% solvent D, and 108–120 min at 100% solvent D.

Data were in positive ion mode acquired via a Q-Exactive mass spectrometer (Thermo Finnigan, San Jose, CA, USA)at a selected mass range of 300–800 mass/charge (m/Z). Moreover, dynamic exclusion was performed for a duration of 40.0 s, and Q-Exactive survey scans were set at resolutions of 70,000 and 17,500 at m/z 200 for HCD spectra. MS/MS data were obtained using the data-dependent acquisition method, targeting the top 10 most abundant precursor ions. The normalized collision energy was set at 30 eV, whereas the underfill ratio on the Q-Exactive mass spectrometer was set as 0.1%.

### Protein identification and quantification

Proteins were identified and quantified using a previously reported protocol [18]. Briefly, the proteins were first searched using the MASCOT search engine (version 2.2.1; Matrix Science, London, UK) embedded in Proteome Discoverer 1.3 (Thermo Electron, San Jose, CA, USA). This was achieved by searching the UniProt database of rat protein sequences (08-2013, downloaded from http://www.uniprot.org/) as well as a decoy database using the following search parameters: monoisotopic mass, peptide mass tolerance of ± 20 ppm, fragment mass tolerance of 0.1 Da; target enzyme of trypsin; and up to two missed cleavages. iTRAQ 8-plex-labeled tyrosine and methionine oxidation were considered variable modifications, whereas carbamidomethylation of cysteine, the N-termini of peptides labeled by iTRAQ 8-plex, and lysine were considered fixed modifications. We set the false discovery rate (FDR) for both protein and peptide identification to less than 0.01, and this threshold was supported by the identification of at least one unique peptide.

### Bioinformatics analyses

We carefully analyzed the significant differentially expressed proteins (DEPs) (*p* < 0.05) and selected those with differential expression ratios above ± 1.2. We then performed hierarchical cluster analysis using Cluster 3.0 and Java TreeView software to determine the value of the identified DEPs in differentiating our two experimental groups. Thereafter, we performed disease and pathway analyses and network generation using ingenuity pathway analysis (IPA) software (QIAGEN, Redwood 185 City, CA) and the general IPA database, which relies on available publications describing the biological mechanisms, interactions and functions of proteins. Here, z-scores were calculated to infer the activation state (“inhibited” or “activated”) of related biological processes. Fisher’s exact test was employed to determine *p*-values and hence predict the likelihood of association among proteins in some datasets. The activation of the resulting biological processes was attributed to chance.

### Western blot assay

Protein expression was measured using Western blot analysis as previously described [18]. Briefly, proteins were extracted by homogenizing thoracic aorta tissues in 500 µL 1× RIPA buffer enriched with protease inhibitors (1 µg/mL phenylmethylsulfonyl fluoride and 1 µg/mL leupeptin). Protein separation was performed by SDS-PAGE (10% TRIS-HCl–SDS gels) using a Mini-PROTEAN Handcast system (BioRad, Hercules, CA, USA). The proteins were resolved and transferred onto a nitrocellulose membrane (0.45 μm, Millipore, Billerica, MA, USA) using standard procedures. The membranes were incubated for 1 h with blocking buffer and then overnight with primary antibodies against glucose-6-phosphate dehydrogenase (G6PD) (Cell Signaling Technology 12,263, Danvers, MA, USA, 1:1000), cysteine- and glycine-rich protein 2 (CSRP2) (Abcam ab178695, Cambridge, UK; 1:1000), tubulin alpha-4 A (TUBA4A) (Sangon D110022, Shanghai, China, 1:1000) and β-Actin (Santa Cruz Biotechnology sc1616, Santa Cruz, CA, 1:1000) at 4 °C overnight. Finally, the membranes were probed with secondary antibody (1:5000) at room temperature for 1 h. The membranes were washed three times, and then the protein band intensities were determined using the Odyssey® Imager system (LI-COR, Lincoln, NE, USA).

### Statistical analysis

GraphPad Prism 6 software (San Diego, CA) was utilized for data analysis. Student’s *t*-test was applied for group comparisons. The data are shown as means ± standard deviations (SDs). *p* < 0.05 was considered statistically significant.

## Results

### Rat model of preeclampsia

**Fig. 1:**
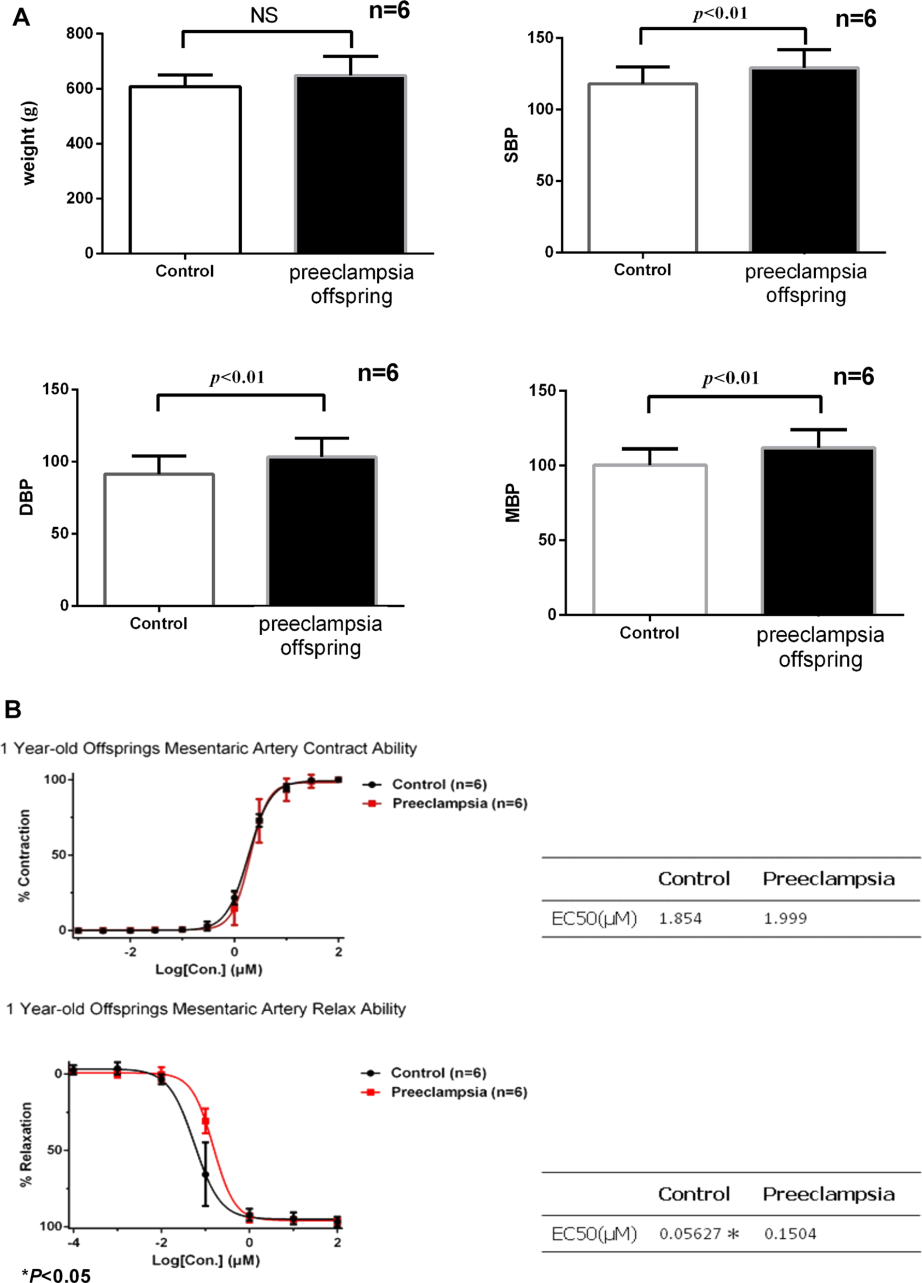
Rat model of preeclampsia. **A** Weight and blood pressure (including SBP: systolic blood pressure; DBP: diastolic blood pressure; MBP: mean blood pressure) of the 1-year-old offspring of preeclampsia model rats. **B** The logEC50 of the contraction and relaxation of mesenteric arteries from the offspring of preeclampsia model rats (1-year-old)

BP was significant difference between the two groups after 1 year (Fig. 1A). No significant changes were observed at 6 months (Additional file 1: Fig. S1), although significant differences were observed in 1-year-old rats. We found no significant difference in the contractile response of mesenteric arteries to Phe between adult rats born to preeclamptic mothers and control rats (Fig. 1B). However, the mesenteric arteries of adult rats born to preeclamptic mothers group exhibited significantly reduced relaxation responses to acetylcholine (an endothelium-dependent vasodilator). The logEC50 values are presented in the table.

**Fig. 2:**
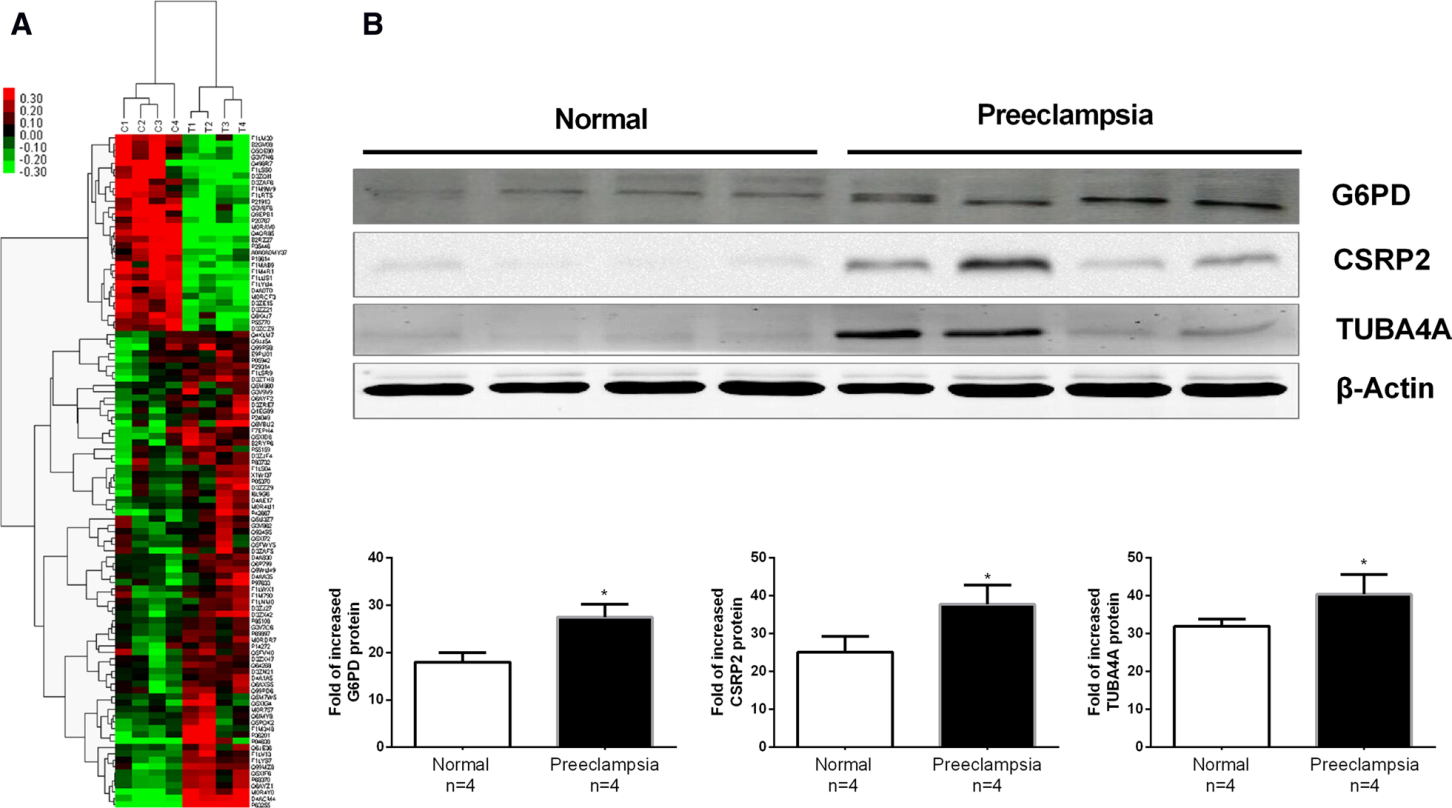
iTRAQ results for the rat thoracic aorta. **A** Hierarchical clustering of DEPs in the rat thoracic aorta. Black, deep green and bright red indicate no change, decreased, and increased, respectively. **B** Confirmation of the differential expression of three proteins. The relative expression levels of three proteins—G6PD, CSRP2 and TUBA4A—in the rat thoracic aorta were confirmed by proteomics. The group data are the means ± SDs (n = 4). p < 0.05 versus controls

We evaluated whether proteomic changes in rat thoracic aorta tissues were related to preeclampsia. Untargeted proteomic analysis identified 1825 nonredundant proteins in rat thoracic aorta tissues (Additional file 2: Table S1). Overall, 106 proteins showed differential expression between the preeclamptic group and control group, of which 75 and 31 proteins were significantly upregulated and downregulated, respectively (Additional file 3: Table S2). Hierarchical clustering of DEPs is illustrated using a heat map (Fig. 2A).

### Validation of protein expression

We validated 3 DEPs of protein expression profiles determined by iTRAQ analysis as described above using Western blot analysis, with a special focus on the correlation between the expression of DEPs and cardiovascular functions. Our blots showed that G6PD, CSRP2, and TUBA4A, which affect cardiovascular function, were significantly expressed (Fig. 2B), which corroborated the findings of iTRAQ analysis.

### Disease and functional analysis

To reveal the function of the identified DEPs, we subjected the expression data to IPA to evaluate the significance of associations. The DEPs were linked to “Disease and Disorder”, as well as “Physiological System Development and Functions”. Moreover, overlapping *p*-values indicated that these proteins were markedly associated with 24 subcategories of “Disease and Disorder” (Additional file 4: Fig. S2) and 19 subcategories of “Physiological System Development and Functions” (Additional file 5: Fig. S3).

**Fig. 3:**
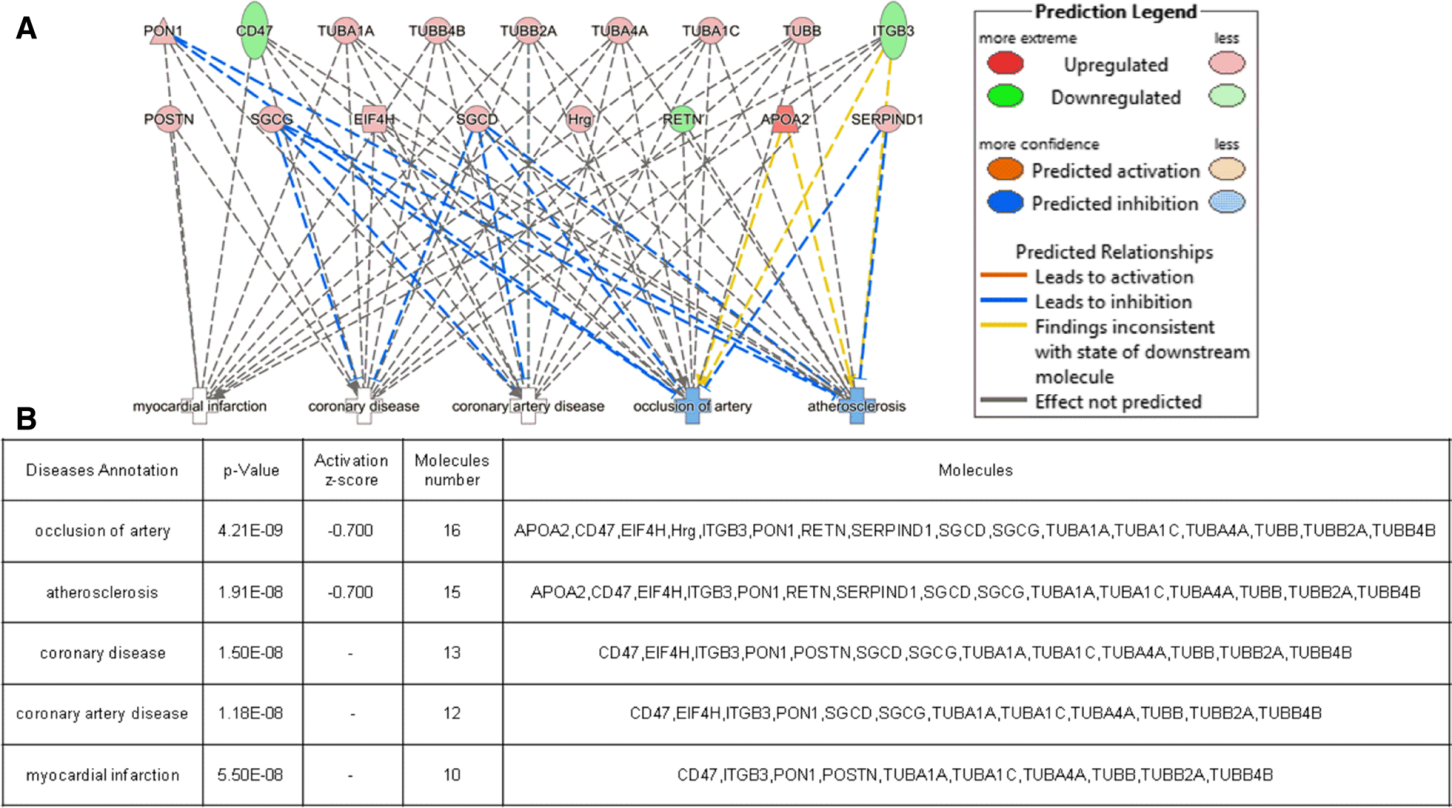
Relationship between specific DEPs and cardiovascular disease based on IPA. Protein names and their temporal expression are outlined in the table. In this cardiovascular disease network, genes or gene products are shown as nodes, and the correlations between two nodes are represented by edges. All edges are supported by at least one publication obtained from the Ingenuity Knowledge database. Red and green nodes indicate upregulated and downregulated proteins, respectively. Interactions in the network as well as the relationships among molecules are summarized on the right side of the figure

**Fig. 4:**
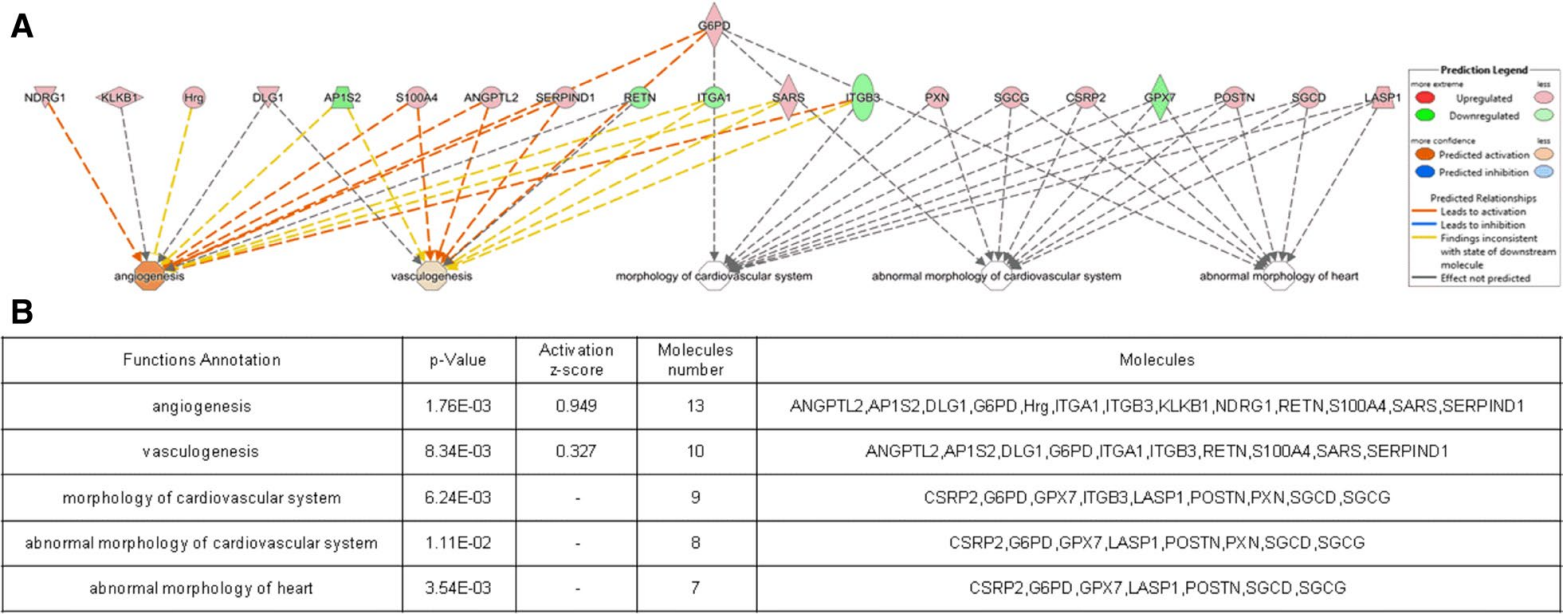
Profile of the downstream effects of specific DEPs associated with cardiovascular function based on iterative analysis. Protein names and their temporal expression patterns are outlined in the table. Genes or gene products are shown as nodes, whereas the biological relationships between two nodes are indicated by edges. All edges are supported by at least one publication in the Ingenuity Knowledge database. Red and green nodes indicate upregulated and downregulated proteins, respectively. Interactions in the network as well as the relationships among molecules are summarized on the right side of the figure

Analysis of the “Disease and Disorder” category further revealed that 22 proteins were associated with cardiovascular disease (Fig. 3), especially occlusion of arteries, atherosclerosis, coronary disease, coronary artery disease and myocardial infarction. Furthermore, 20 proteins were found to be linked to cardiovascular functions (Fig. 4), especially angiogenesis, vasculogenesis, abnormal morphology of the heart, abnormal morphology of the cardiovascular system, and morphology of the cardiovascular system.

### Upstream analysis

**Fig. 5:**
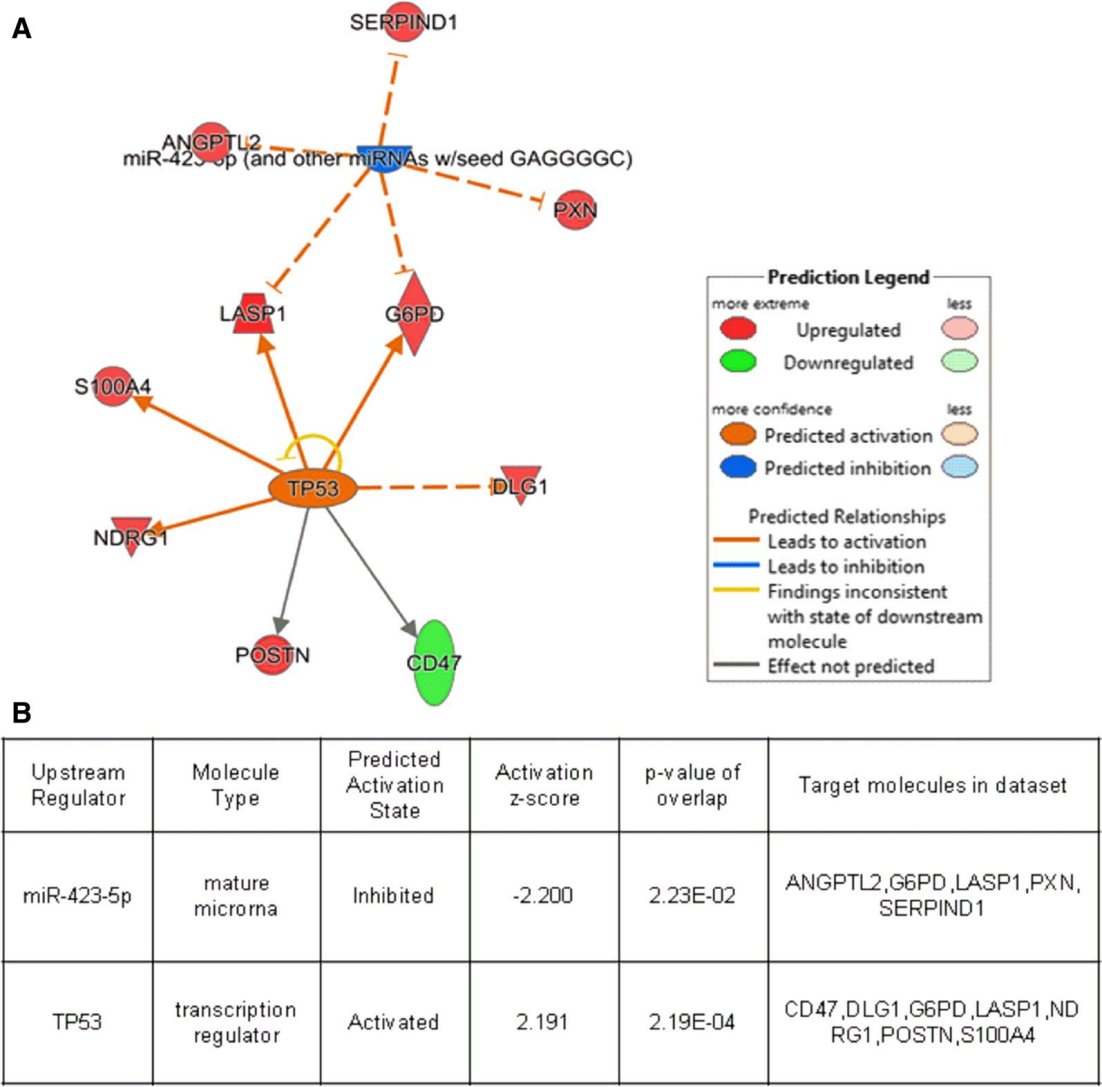
Upstream analysis by IPA based on 20 DEPs were related to cardiovascular functions. The colored nodes represent our input proteins. Red: upregulated proteins. Green: downregulated proteins

According to IPA, the term “upstream regulator” is used to refer biomolecules with the potential to influence the expression of another biomolecule. Previous studies have shown that upstream regulators can be drugs, chemicals, kinases, receptors, microRNAs, cytokines, or transcription factors. The results of the present study revealed that of the 20 proteins associated with cardiovascular functions, miR-423-5p and TP53 were upstream regulators. miR-423-5p inhibited the expression of ANGPTL2, G6PD, LASP1, PXN and SERPIND1, while TP53 mediated the activation of CD47, DLG1, G6PD, LASP1, NDRG1, POSTN and S100A4 expression (Fig. 5).

## Discussion

Proteomics, which refers to the functional analysis of all proteins expressed in an organism, has been widely used to complement other functional genomics approaches. Bioinformatics analysis of these datasets can lead to the development of a comprehensive gene function database, which provides useful tools for establishing and testing research hypotheses. Previous studies have shown that mass spectrometry (MS)-based proteomics approaches have are highly valuable for the medical field [20]. Of note, iTRAQ is used to characterize proteins. In our previous studies, we determined the protein profiles of human umbilical arteries from preeclamptic, in vitro fertilization (IVF) and ovarian hyperstimulation syndrome (OHSS) patients [18, 21, 22]. Interestingly, our results identified proteins that were differentially expressed in these patients relative to controls and are highly correlated with cardiovascular physiology. Previous studies aimed to reveal the events leading to infant cardiovascular diseases. In this study, we analyzed thoracic aortas from adult rats born to preeclamptic dams and identified proteins related to cardiovascular function.

Herein, the association of protein expression with disease and function was examined. We found that 22 DEPs were significantly associated with cardiovascular diseases. Additionally, we found that occlusion of arteries and atherosclerosis were the disorders that were the most significantly associated with exposure of offspring to preeclampsia, as evidenced by their significant positive correlation with the expression of 16 DEPs. Among these DEPs, the protein encoded by the APOA2 transgene affects the development of atherosclerotic lesions in a mouse model [23, 24], and the SGCG protein decreases coronary artery spasm in mice [25]. Knockout of the SERPIND1 gene in mice increases the formation of atherosclerotic plaques in the mouse aortic root [26]. Knockout of the Pon1 gene exacerbates atherosclerosis in mice [27]. Mutation of the human EIF4H gene is associated with coronary artery disease in humans [28]. In human peripheral blood monocytes, the expression of CD47 protein in detergent-resistant membranes is linked to myocardial infarction and coronary artery disease in humans [29]. In mice, knockout of the ITGB3 gene exacerbates atherosclerosis of the aorta [30].

In the analysis of cardiovascular function, we found that 20 proteins were related to cardiovascular function, and that angiogenesis was the most affected function. Among these proteins, mouse Hrg relieves angiogenesis of blood vessels [31]. A dominant negative protein fragment (306–423) containing a DNA binding domain from the mouse AP1S2 protein alleviates angiogenesis in the mouse ear [32]. The NDRG1 protein increases angiogenesis in tumors derived from NUGC-3 cells from the mouse subcutaneous fascia [33]. G6PD modulates VEGF-mediated angiogenesis [34], and interference with human SARS mRNA by an siRNA increases the sprouting of cultured HUVECs [35]. Human DLG1 is involved in the proliferation of endothelial cells [36], and rat ITGB3 increases angiogenesis of blood vessels [37]. In mice, knockout of Integrin Subunit Alpha 1 (ITGA1) decreases angiogenesis of blood vessels in tumors [38]. The ANGPTL2 protein increases angiogenesis in mouse [39], and the S100A4 protein can act as an angiogenic factor [40]. The KLKB1 protein is involved in angiogenesis in mammals [41]. Based on these findings, it is evident that ectopic expression of these proteins can exacerbate susceptibility to cardiovascular disease. However, the precise relationship between these proteins and the fetal origins of adult cardiovascular disease remains unclear, necessitating further investigation.

Upstream regulator analysis (UPA) has been extensively used to detect upstream regulators that control gene expression changes across experimental datasets. Functionally, IPA allows prediction of activated or inhibited upstream regulators, thereby revealing upregulated and downregulated genes in a dataset. Knowledge of this regulatory cascade allows accurate elucidation of biological activities that may occur in tissues or cells. During upstream analysis of cardiovascular function, miR-423-5p was implicated in the inhibition of G6PD and LASP1 activity, while TP53 was shown to activate G6PD and LASP1 expression. On the other hand, miR-423-5p is a potential biomarker for the diagnosis and prognosis of heart failure [42], whereas TP53 has been associated with changes in DBP [43]. Therefore, upstream analysis represents a feasible approach for future studies.

Previous proteomics studies targeting the human umbilical artery have reported that the DEPs identified in the present study play key roles in the cardiovascular system. Together, the results indicate that preeclampsia could lead to vascular dysfunction, especially angiogenesis, in offspring. Some of the DEPs are related to cardiovascular disease. Therefore, we can conclude that preeclampsia could affect the cardiovascular system of offspring.

## Conclusions

In conclusion, 24 DEPs were related to cardiovascular disease, and 22 DEPs were described to be associated with cardiovascular functions, especially atherosclerosis and angiogenesis. It is crucial to determine whether the DEPs identified herein are the cause or the effect of cardiovascular dysfunction in the offspring of preeclamptic mothers. In particular, unraveling the relationship between cardiovascular function-relate proteins and cardiovascular processes could provide useful insight into the pathological events underlying vascular diseases.

## Supplementary Information

**Additional file 1: Fig. S1.** Rat model of preeclampsia. (A) Weight and blood pressure (including SBP: systolic blood pressure; DBP: diastolic blood pressure; MBP: mean blood pressure) of the half-year-old offspring of preeclampsia model rats. (B) The logEC50 of the contraction and relaxation of mesenteric arteries from the offspring of preeclampsia model rats (half-year-old).

**Additional file 2: Table S1.** A total of 1825 nonredundant proteins in the thoracic aorta were identified with high confidence.

**Additional file 3: Table S2.** A total of 106 proteins showed differential expression between the preeclampsia group and the control group/ The expression of 75 proteins was elevated, whereas that of 31 proteins was decreased.

**Additional file 4: Fig. S2.** The DEPs were strongly related to 24 subcategories of “Disease and Disorder”.

**Additional file 5: Fig. S3.** The DEPs were strongly related to 19 subcategories of “Physiological System Development and Functions”.

## Data Availability

The mass spectrometry proteomics data have been deposited to the ProteomeXchange Consortium (http://proteomecentral.proteomexchange.org) via the iProX partner repository [44] with the dataset identifier PXD024811.

## Abbreviations

iTRAQ: Isobaric Tag for Relative and Absolute Quantitation
IPA: Ingenuity pathway analysis
SCX: Strong cationic-exchange chromatography
FDR: False discovery rate
DEPs: Differentially expressed proteins
BP: Blood pressure
l-NAME: *N*-omega-nitro-l-arginine methyl ester
