# Supplementary material for: iTRAQ-based quantitative proteomic analysis of thoracic aortas from adult rats born to preeclamptic dams
Source: Clin Proteomics. 2021 Aug 21;18:22. doi: 10.1186/s12014-021-09327-9 (PMC8379584; doi:10.1186/s12014-021-09327-9)
Supplement: Supplementary file 1 — Additional file 1: Fig. S1. Rat model of preeclampsia. (A) Weight and blood pressure (including SBP: systolic blood pressure; DBP: diastolic blood pressure; MBP: mean blood pressure) of the half-year-old offspring of preeclampsia model rats. (B) The logEC50 of the contraction and relaxation of mesenteric arteries from the offspring of preeclampsia model rats (half-year-old). [file 12014_2021_9327_MOESM1_ESM.pdf]

A

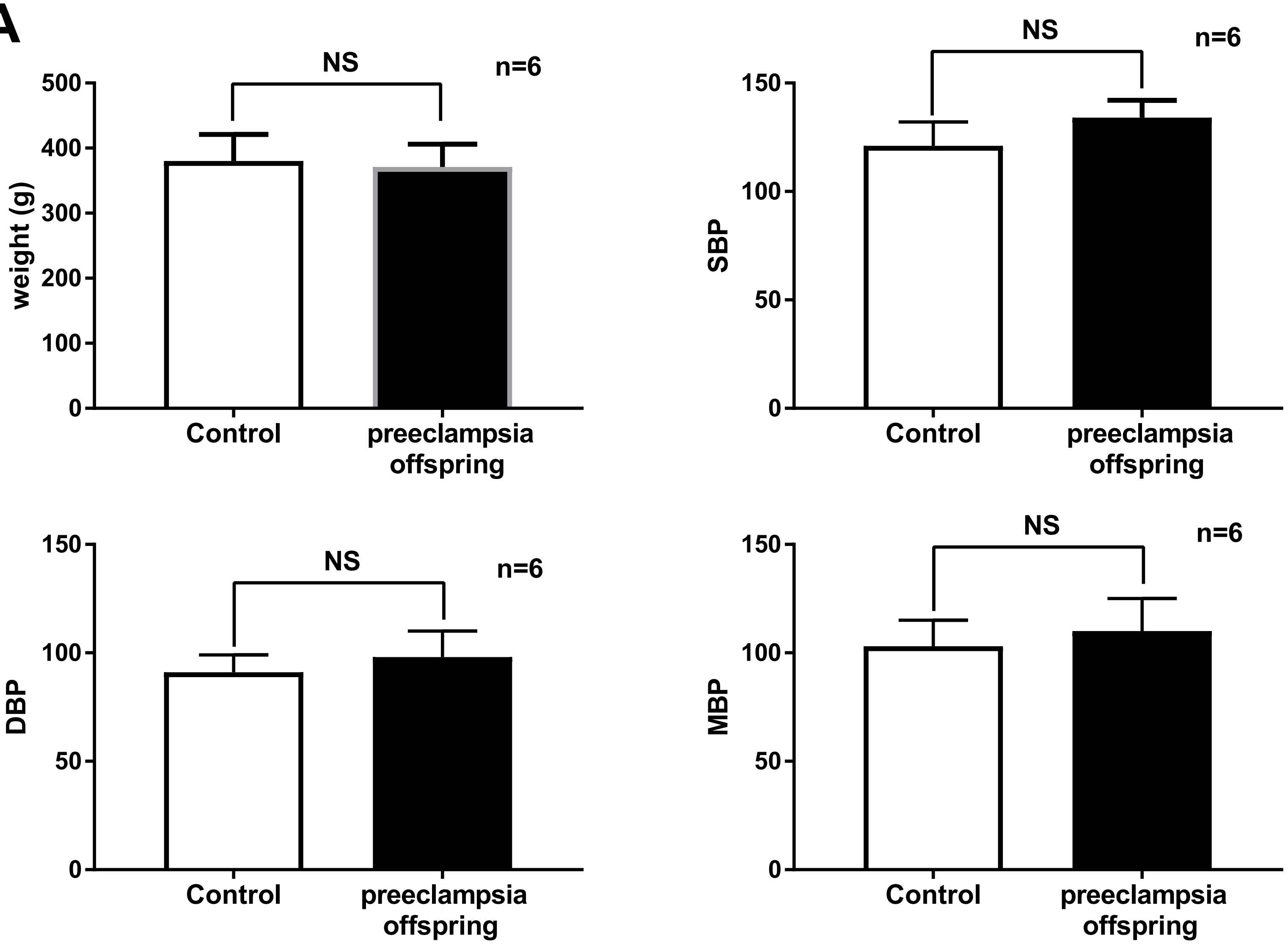

B

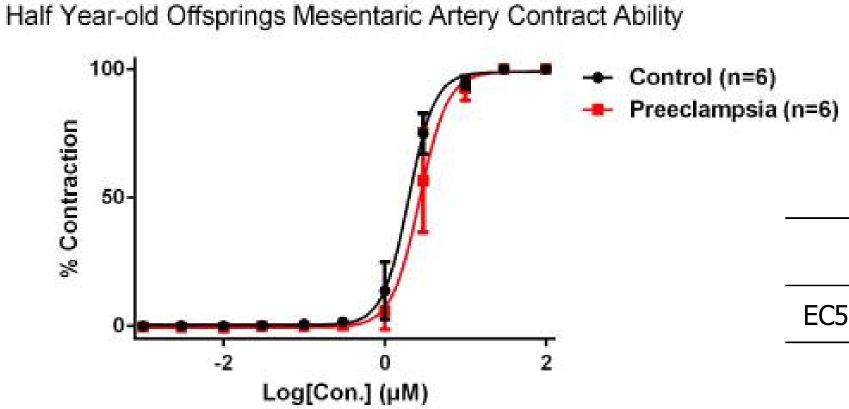

	Control	Preeclampsia
EC50(μM)	1.974	2.699

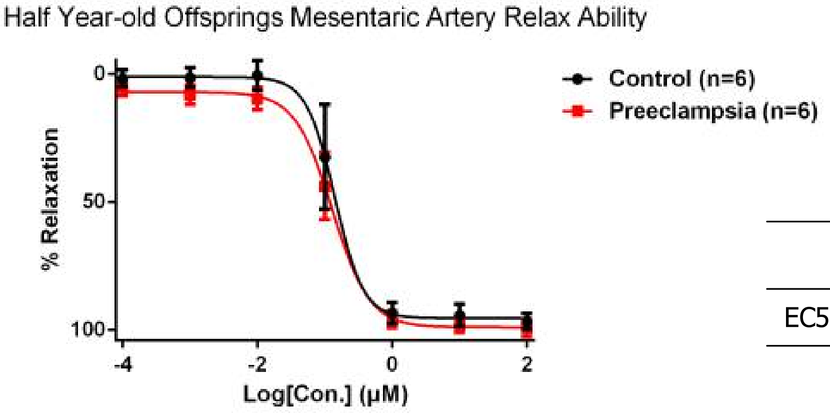

	Control	Preeclampsia
EC50(μM)	0.1408	0.1290
