# Supplementary figures and images for: iTRAQ-based quantitative proteomic analysis of thoracic aortas from adult rats born to preeclamptic dams
Source: Clin Proteomics. 2021 Aug 21;18:22. doi: 10.1186/s12014-021-09327-9 (PMC8379584; doi:10.1186/s12014-021-09327-9)

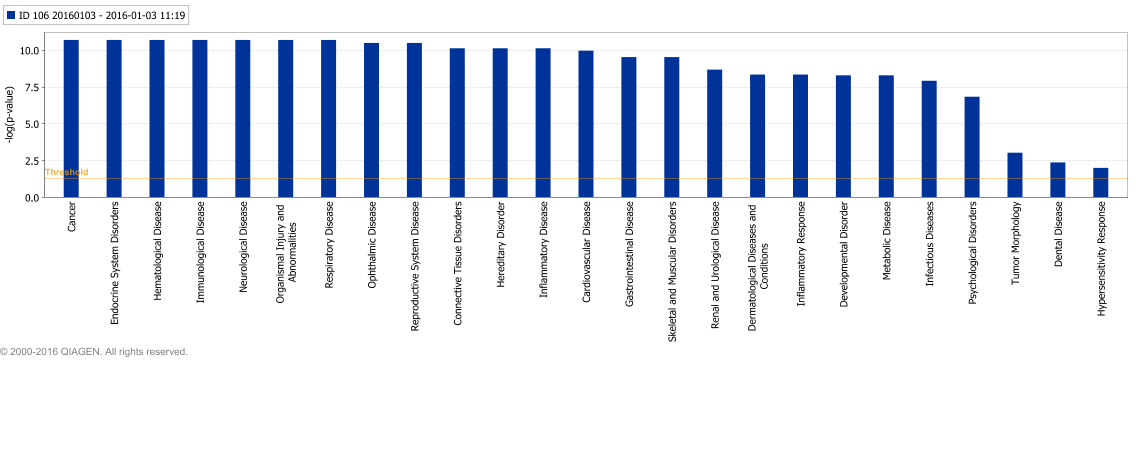

Supplement: Supplementary file 4 — Additional file 4: Fig. S2. The DEPs were strongly related to 24 subcategories of “Disease and Disorder”. [file 12014_2021_9327_MOESM4_ESM.tif]

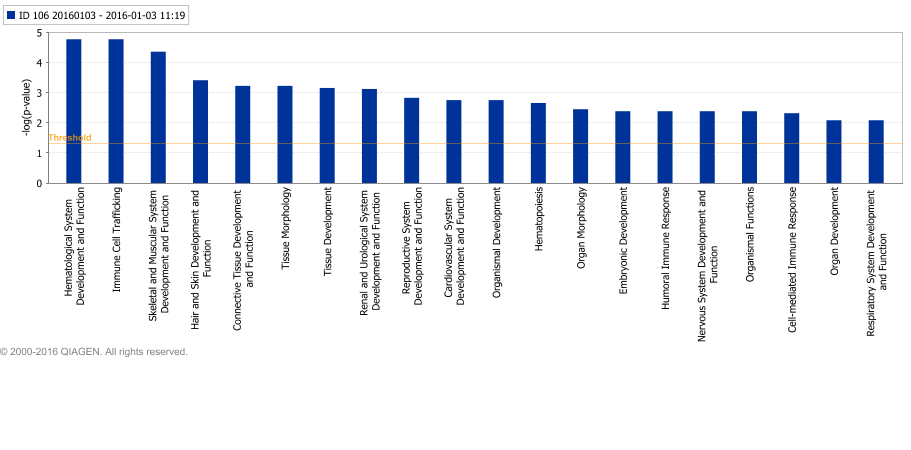

Supplement: Supplementary file 5 — Additional file 5: Fig. S3. The DEPs were strongly related to 19 subcategories of “Physiological System Development and Functions”. [file 12014_2021_9327_MOESM5_ESM.tif]
